# Reduction in blood pressure and serum lipids by lycosome formulation of dark chocolate and lycopene in prehypertension

**DOI:** 10.1002/fsn3.169

**Published:** 2014-09-17

**Authors:** Ivan M Petyaev, Pavel Y Dovgalevsky, Natalia E Chalyk, Victor Klochkov, Nigel H Kyle

**Affiliations:** 1 Lycotec LtdGranta Park Campus, Cambridge, CB21 6GP, United Kingdom; 2 Institute of Cardiology12 Chernyshevskogo Str, Saratov, 410028, Russia

**Keywords:** Dark chocolate, lycopene, prehypertension, serum lipids

## Abstract

Twenty-nine healthy volunteers aged 47–69 years old were randomly assigned to a 28-day oral intake of different dark chocolate (DC) formulations. The main group received daily 30 g of proprietary lycopene-containing (L-tug) lycosome formulation of DC with enhanced bioavailability of cocoa flavanols. Two control groups daily consumed either 30 g of regular DC alone or along with 7 mg of lycopene, which corresponds to the amount of lycopene ingested with L-tug formulation. It was found that L-tug was more efficient in reducing diastolic blood pressure (mean value of −6.22 mmHg, 95% CI: 5.00, 8.00) when compared with the regular DC group (−3.00 mmHg, *P* < 0.05) or the group which ingested the DC and lycopene as two separate formulations (mean reduction of −4 mmHg, 95% CI: 2.47, 6.00, *P* = 0.0262). Only marginal superiority for L-tug formulation in the reduction in systolic blood pressure was seen. However, the L-tug formulation was the only formulation of DC which affected serum lipids. There was a reduction in total cholesterol (from median 228.00 mg/dL [95% CI: 206.2, 242.5] to 187.00 mg/dL [95% CI: 166.2, 202.2, *P* < 0.05]) with corresponding decline of low-density lipoprotein (LDL) cholesterol (from a median of 166.00 mg/dL [95% CI: 130.8, 177.0] to 151.00 mg/dL [95% CI: 122.8, 167.4; *P* < 0.05]) at the end of the intervention period. Similar decline was seen in serum triglycerides (*P* < 0.05). Serum high-density lipoprotein (HDL) cholesterol, glucose levels, and C-reactive protein (CRP) values remained statistically unchanged in all study groups throughout the intervention period. A superior biological activity of the L-tug lycosome formulation of DC extending beyond its antihypertensive effect to lipid-lowering ability opens up new possibilities for the use of DC for health purposes helping to reduce daily caloric intake without compromising on the health benefits of DC consumption.

## Introduction

Oxidative stress and the subsequent accumulation of reactive oxygen species represent a basic unifying mechanism behind atherosclerosis development under different pathological conditions and creates a major rationale for the use of antioxidants in the prevention and treatment of cardiovascular disease (Singh and Jialal 2006; Lönn et al. 2012). There is a growing body of both epidemiological and clinical evidence that cocoa flavanols (catechins and epicatechins) and cocoa alkaloids (theobromine and theophylline) can be used effectively for the prevention and treatment of cardiovascular disease (Flammer et al. 2012; Stote et al. 2012; Cicero and Borghi 2013). These claims have significant support from multiple in vivo and in vitro studies describing the antioxidant effect of cocoa flavanols and alkaloids resulting in modulation of nitric oxide production and cyclic adenosine monophosphate (cAMP) turnover (Park 2005; Gülçin 2012; Fernández Vallinas et al. 2013). However, cocoa-derived products remain extremely underrepresented in the modern human diet. In general, cocoa flavanols contribute only 0.1% of the total daily flavonoid intake even in developed countries with an established cocoa-consuming culture (Ilow et al. 2012). Therefore, even a moderate increase in the habitual intake of cocoa flavanols seems to hold substantial promise for human health. Indeed, meta-analysis studies show that the health effects of cocoa flavanols, in particular, their effect on systemic blood pressure reflect the amount of flavanols ingested (Ellinger et al. 2012). However, a simple increase in the habitual intake of commercially available cocoa-based products may not guarantee for the appearance of the anticipated health benefits of flavanols due to poor intestinal absorption of catechins/epicatechins from natural cocoa products (Li and Hagerman 2013). More disturbingly, commercially available cocoa-based products including dark chocolate do not have strict manufacturing requirements or packaging/labeling specifications disclosing phenolic content or recommended daily intake (Rusconi and Conti 2010). No official analytical methodology for the quantification of phenolic compounds has been introduced yet (Rusconi and Conti 2010). Therefore, any public health recommendation relating to the use of cocoa polyphenols for health-related reasons remains impossible for the moment. Scrupulous investigation of the health benefits of cocoa polyphenols in the form of polyphenol-rich foods or isolated phenolic compounds is an urgent task for modern food science (Habauzit and Morand 2012). The development of new nutraceutical formulations of dark chocolate with high bioavailable cocoa flavanols holds promise among various options.

In the present paper, we report the reduction in systemic blood pressure and serum lipids (cholesterol and triglycerides) in prehypertensive clinically healthy volunteers after ingestion of a proprietary lycopene-containing formulation of dark chocolate L-tug™, which has been developed by Lycotec Ltd (Cambridge, UK) using lycosome technology (Petyaev 2012).

## Materials and Methods

### Study protocol

The study was conducted by Lycotec Ltd at the Institute of Cardiology, the Ministry of Health of the Russian Federation (Saratov, Russia). The protocol was approved by the local Ethics Committee and registered (ACTRN12613000966796). All volunteers were aware of the purpose of the study and signed a written consent form regarding their participation. All volunteers underwent physical and laboratory examinations, and their medical history were evaluated. Prehypertension was defined according to the guidelines of the USA Joint National Committee, JNC 7 (Chobanian et al. 2003), as a state of having systolic blood pressure between 130 and 139 mmHg, and diastolic blood pressure between 80 and 89 mmHg. The prehypertensive individuals were further screened for serum lipid levels: total cholesterol, low- and high-density lipoproteins (LDL and HDL), and triglycerides. Those who had low-to-moderate hypercholesterolemia (defined as elevated total serum cholesterol from 200 to 250 mg/dL) were recruited for the trial.

### Subjects and inclusion criteria

From the selected pool of volunteers, three randomized groups from the total of 34 people were formed (Table1). Five volunteers were not able to complete the trial due to dark chocolate intolerance or for nonhealth-related reasons. Dropouts were replaced with other eligible volunteers from the preselected pool. Major inclusion criteria were as follows: Caucasian male or female subjects 45–70 years old; sustained resting blood pressure (systolic – 130–139 mmHg, diastolic 80–89 mmHg); absence of concomitant intake of antihypertensive, lipid-lowering, or any other cardiovascular drugs; and elevated total serum cholesterol level (200–250 mg/dL). The exclusion criteria were: inability to comply with the study protocol, severe medical conditions (hepatitis, pancreatitis, uncontrolled diabetes, cancer, recent cardiovascular events, tuberculosis, etc.). The volunteers were asked to refrain from consumption of cocoa- and tomato-based products for 10 days before beginning the trial. After completion of the run-in period patients were given the trial dark chocolate products.

**Table 1:** Baseline characteristics of the enrolled volunteers (mean ± SD)

	Study groups		
Variable	Dark chocolate	Dark chocolate/lycopene	Dark L-tug chocolate
Number of patients (*n*)	10	10	9
Age (years)	54.02 ± 4.92	56.7 ± 7.68	54.77 ± 6.26
Gender			
Male (%)	40	50	55.5
Female (%)	60	50	44.5
Smokers (%)	30	40	33.3
BMI	28.42 ± 2.71	27.18 ± 4.57*	27.95 ± 4.64*
Pulse rate	70.1 ± 3.63	67.5 ± 4.57*	69.89 ± 4.61*
Blood pressure			
Systolic	135.2 ± 2.69	131.4 ± 2.22*	137.33 ± 1.87*
Diastolic	84.2 ± 2.85	84.70 ± 2.40*	81.55 ± 2.12*
CRP	5.08 ± 1.33	4.62 ± 1.49*	4.22 ± 1.80*
Cholesterol, mg/dL	217.5 ± 10.67	222.30 ± 13.27*	230.56 ± 14.10*
Triglycerides, mg/dL	165.00 ± 16.69	159.10 ± 17.89*	161.33 ± 33.15*
HDL, mg/dL	40.50 ± 2.01	40.10 ± 2.02*	40.00 ± 2.34*
LDL, mg/dL	155.2 ± 14.54	157 ± 14.11*	157.11 ± 18.87*
Glucose, mmol	5.48 ± 0.97	5.5 ± 0.94*	5.25 ± 0.74*

Volunteers were selected, randomized into groups. Mean and SD values for different randomization criteria were calculated as described in the “Materials and Methods” section.

* Insignificant changes with *P* > 0.05 as compared to the chocolate group.

### Trial design

After the run-in period, volunteers from each group received a 2-week supply of 14 blind control chocolate bars. Volunteers from the first group, designated throughout the study as “DC” group, received their 2-week supply of regular dark chocolate. Volunteers from the second group, named as the “DC+LC” group, were given regular dark chocolate bars plus 14 capsules of lycopene. In the third group (“L-tug group”), volunteers received a 2-week supply of 14 blind L-tug composite chocolate bars containing lycopene. In all groups, the participants were not aware of specifications of dark chocolate products.

All volunteers were instructed to ingest one chocolate bar once a day after the main meal. Volunteers in the second group were asked to ingest the lycopene capsule (referred as a food supplement) at the same time as the dark chocolate. All volunteers were instructed to keep their chocolate packaging and bring it to the following clinical visit. After verification of compliance, the packaging was exchanged for a fresh 14-day supply of products. The trial lasted 4 weeks. In the middle of the trial (14th day), when volunteers received the second batch of products, they underwent clinical examination and blood test. A similar examination and blood test were performed at the end of the trial (28th day).

### Products

#### Dark chocolate

Dark chocolate bars (30 g) with 85% cocoa from Green & Black's Organic (East Hanover, NJ) were used in all groups of the trial. The chocolate was melted, treated, and tempered in exactly the same way for all groups of the study regardless of the addition of lycopene. Nutritional parameters, catechin, theobromine, and caffeine contents of Green & Black's dark chocolate are available from the manufacturer.

#### L-tug™ formulation of dark chocolate

L-tug™ formulation of dark chocolate is a proprietary lycosome formulation of dark chocolate (Lycotec Ltd) with an enhanced bioavailability of cocoa flavanols in which cocoa-derived bioactive compounds are protected from oxidation by a lycopene layer and embedded into cocoa butter micelles of the chocolate matrix (I. M. Petyaev, D. Pristenskiy, T. Bandaletova, N. E. Chalyk, Victor Klochkov, and N. H. Kyle, under review; Carotenoid Particles and Uses Thereof; Cocoa-Based Food Products). The final concentration of lycopene in the composite lycosome formulation was 7 mg per 30 g piece of chocolate. The lycopene used was in the form of tomato oleoresin from Lycored Inc (New Jersey, NJ) and contained 97% of *trans*-isomers and 3% of *cis*-isomers.

#### Lycopene

Lycopene control capsules containing 7 mg of lycopene (Lycored Inc) were made from the same batch of tomato oleoresin that was used for the preparation of the lycosome formulation of dark chocolate.

### Methods

#### BMI, pulse rate, and blood pressure

Body mass index (BMI) was calculated as described elsewhere. Pulse rate, systolic, and diastolic blood pressure were measured three times in the left arm of seated volunteers after 15 min of rest. The time between measurements was no less than 2 min. The mean value for each parameter was calculated. All parameters were measured in the morning between 8 and 10.

#### Blood collection

Blood was collected from the arm veins of the volunteers in the morning after fasting. Serum was separated from the clotted mass by centrifugation and aliquots were stored at −80°C prior to analysis.

#### Laboratory parameters

Total cholesterol (TC), triglycerides (TG), HDL/LDL cholesterol, glucose, and C-reactive protein (CRP) were measured using a Biosystem A25 automated analyzer (Applied Biosystems, Grand Island, NY) using BioSys kits and calibrators.

### Statistics

For the assessment of normally distributed parameters, the Shapiro–Wilk method was used. Student's *t*-test was then applied both for paired and unpaired samples. Between-group differences at one time point were evaluated by the Wilcoxon–Mann–Whitney test (continuous variables) and Fisher's exact test (categorical variables). Data analysis was performed using Stata (College Station, TX) SE, version 12.1. All statistical tests were two sided and statistical significance level alpha was set at 0.05 for the analysis.

## Results

As can be seen from Table1, the study was conducted using similar groups of volunteers. No statistically significant differences were seen in age, gender, BMI, or smoking status of the individuals enrolled in the three major groups of the study. Moreover, no dissimilarities in the baseline blood pressure, pulse rate, lipid profile, and blood glucose levels were seen among the individuals suggesting acceptable randomization.

Upon completion of the 1-month study period, some small within-group fluctuations in the body mass, of the three major groups of the study, had fallen behind the cutoff value of statistical significance accepted in our study (results not shown). There were no changes in the BMI to be reported.

However, as can be seen from Table2, the volunteers from all three groups of the study had a statistically significant reduction in systemic blood pressure at the end of the 4-week interventional period. The groups differed in the magnitude of blood pressure reduction. In particular, lycosome formulation of dark chocolate (L-tug group) caused more significant reduction in diastolic blood pressure (mean reduction of −6.22 mmHg) in comparison with the regular dark chocolate group (−3.00 mmHg, *P* < 0.05), or the group of individuals who ingested the dark chocolate and lycopene as two separate formulations (−4 mmHg, *P* < 0.05). As for the regulation of systolic blood pressure, there was a statistically significant reduction in posttreatment mean values in all groups of the study (DC group – 6.5 mmHg, DC+LC group −6.00 mmHg, L-tug group −8.00 mmHg, *P*_1,2.3_ < 0.05 as compared to pretreatment values). However, the between-group differences at the end of the interventional period were insignificant, showing just marginal superiority (*P* = 0.0651) of L-tug formulation when compared with regular dark chocolate. Changes in the systemic blood pressure in the middle of the interventional period were less consistent and are not reported here.

**Table 2:** Group median values and 5–95% CIs for blood pressure in volunteers treated with different dark chocolate formulations

	Means with 5–95% CI for blood pressure (mmHg)					
	Before treatment		After treatment		Significance	
Group	Systolic	Diastolic	Systolic	Diastolic	*P*_systolic_	*P*_diastolic_
DC (*n* = 10)	135.5 (132.47, 137.52)	84.5 (81.00, 87.00)	128.00 (125.00, 132.00)	80.50 (78.47, 84.00)	<0.05	<0.05
DC+LC (*n* = 10)	130.50 (130.00, 133.10)	84.50 (82.47, 87.52)	125.00 (124.47, 127.53)	81.50 (79.00, 82.52)	<0.05	<0.05
L-tug (*n* = 9)	137.00 (134.80, 139.60)	81.00 (80.00 85.20)	130.00 (125.8, 132.6)	74.00 (72.4, 79.6)	<0.05	<0.05

Volunteers were selected, randomized into groups, and entered the dietary trial. Blood pressure values were obtained for each individual volunteer before and after 28-day dark chocolate product consumption. Median values and confidence intervals were calculated as described in the “Materials and Methods” section.

Furthermore, there were some measurable differences in serum lipids during the intervention period (Table3). In particular, total cholesterol level declined after 2 weeks of L-tug dark chocolate consumption (reduction in median by −34 mg/dL) and remained reduced at the end of the intervention by −41 mg/dL as compared to the median pretreatment value. In contrast, total cholesterol level remained basically unchanged in volunteers treated with either the dark chocolate alone or combined intake of dark chocolate and lycopene.

**Table 3:** Median values with 5% and 95% CIs for laboratory parameters

Parameters in groups	Pretreatment			2 weeks			4 weeks		
	Median	5/95% CI	*P*	Median	5/95% CI	*P*	Median	5/95% CI	*P*
Total cholesterol									
DC	218	202.5/231.1	–	216	201.5/228.2	>0.05	210	198.2/228.8	>0.05
DC+LC	220	206.2/242.5	–	220	206.9/239	>0.05	214.5	203.7/228.5	>0.05
L-Tug	228	206.2/242.5	–	194	171.4/214.2	<0.05	187	166.2/202.2	<0.05
LDL									
DC	157.5	133.4/173.1	–	155	134.5/171.0	>0.05	166	138.5/168.7	>0.05
DC+LC	155	140.4/179.7	–	153.5	139.3/177.1	>0.05	153.5	138.7/171.6	>0.05
L-Tug	166	130.8/177	–	162	126.2/171.8	>0.05	151	122.8/167.4	<0.05
HDL									
DC	40.5	38.4/43.6	–	40.5	39/43.65	>0.05	41	39/43.1	>0.05
DC+LC	40	37.4/43.1	–	40.5	39/43	>0.05	40.5	38.4/42	>0.05
L-Tug	40	36.4/42.6	–	41	37.8/42.6	>0.05	42	39.8/43	>0.05
Triglycerides									
DC	169	139.6/182.5	–	167.5	148.7/183.6	>0.05	163	141.4/176.8	>0.05
DC+LC	163	129.6/178.7	–	160.5	134.7/178.2	>0.05	157	138.7/173.6	>0.05
L-Tug	168	116.2/199.6	–	159	101.8/192.4	<0.05	157	138.7/173.6	<0.05
CRP									
DC	5.35	2.9/6.5	–	n/d	n/d	˜	4.9	3.3/6.0	>0.05
DC+LC	4.9	1.8/6.7	–	n/d	n/d	˜	5	3.2/4.8	>0.05
L-Tug	4.7	2.4/6.2	–	n/d	n/d	˜	4.4	3.0/6.1	>0.05
Glucose									
DC	5.7	3.9/6.6	–	5.8	3.7/6.3	>0.05	5.3	3.7/6.3	>0.05
DC+LC	5.8	4.0/6.4	–	5.7	3.8/6.7	>0.05	5.2	4.5/6.1	>0.05
L-Tug	5.3	4.4/6.2	–	4.9	4.0/6.1	>0.05	5	4.0/5.9	>0.05

Volunteers were selected, randomized into groups, and entered the dietary trial. Serum specimens were collected and analyzed for lipid concentration for each individual volunteer before, after 14th and 28th days of dark chocolate product consumption. Median values and confidence intervals were calculated as described in the “Materials and Methods” section. C-reactive protein values were measured only before and after the interventional period (n/d, not determined).

A similar pattern was found for LDL cholesterol levels. No changes in LDL cholesterol were seen in “DC” and “DC+LC” groups. However, volunteers in the “L-tug” group had a significant reduction in LDL cholesterol upon completion of the intervention period (reduction in median by −15 mg/dL as compared to pretreatment values). Another feature of the lipid-lowering effect of L-tug chocolate was observed when total triglyceride levels were analyzed. We found that serum triglyceride level was noticeably reduced in the L-tug group following 2- (average reduction by – 10.77 mg/dL) and 4-week intervention (average reduction by −23.22 mg/dL) as compared to the pretreatment values. No corresponding changes in serum triglyceride levels were seen in either the “DC” or “DC+LC” groups.

Serum HDL levels, CRP, and glucose concentration remained unchanged in all study groups throughout the intervention period (Table3).

## Discussion

In the present paper, we report that the proprietary lycosome formulation of dark chocolate containing lycopene (L-tug) has a superior ability to reduce systemic diastolic and, to some extent, the systolic blood pressure in prehypertensive individuals as compared to regular dark chocolate or a combination of dark chocolate and lycopene ingested as two separate formulations. Moreover, consumption of the L-tug chocolate is accompanied by a reduction in serum levels of total cholesterol, LDL, and triglycerides, an effect which was not seen in the other two study groups. It is also important to note that the lipid-lowering effect of the L-tug chocolate took place in a time-dependent manner, occurring initially after 2 weeks of product consumption and reaching a peak value after 4 weeks of intake.

Our study has some significant limitations. First, a larger number of participants need to be involved in future trials. Second, the dose–response characteristics of L-tug dark chocolate need to be evaluated. Finally, and most importantly, additional in vivo and possibly in vitro studies have to be performed to elucidate the mechanisms behind the superior functional activity of L-tug lycosome-formulated dark chocolate.

In a general sense, our results are in agreement with multiple reports on antihypertensive properties of cocoa-based products and their major constituent — cocoa flavanols. It is believed that even short-term consumption of a cocoa-rich product may have a measurable positive impact on a number of cardiovascular parameters including arterial vasodilation, platelet aggregation, myocardial reperfusion, and systemic blood pressure (Belz and Mohr-Kahaly 2011; Haber and Gallus 2012). In many studies, dark chocolate consumption led to a small (3–6 mmHg) but highly reproducible decrease in both systolic and diastolic blood pressure (Desch et al. 2010). Similarly, changes in serum lipid profile in dark chocolate consumers have been reported by some authors. It has been shown that dark chocolate and cocoa polyphenols can normalize serum lipids by decreasing LDL levels (Jia et al. 2010) and increasing HDL in plasma (Mursu et al. 2004).

Therefore, our results regarding the blood pressure-reducing effect of dark chocolate and its lipid-lowering action, at first sight, do not add anything new to the medical phenomenology of dark chocolate consumption. However, a most important thing arises and becomes clear from our between-group statistical analysis. Despite a similar amount of dark chocolate having been ingested in all groups of the study, lycosome L-tug formulation of dark chocolate caused the most profound decrease in systemic blood pressure. Moreover, the lipid-lowering effect of dark chocolate observed in our study was clearly attributable to the L-tug formulation only. It is well known that lycopene is a powerful inhibitor of 3-hydroxy-3-methyl-glutaryl-CoA reductase (HMG-CoA reductase), a rate-limiting hepatic enzyme of cholesterol biosynthesis (Palozza et al. 2012). However, it is clear from our results that mere ingestion of the dark chocolate and lycopene as two separate nutraceuticals was not sufficient enough to induce any positive changes in serum lipid profile. Moreover, traditionally, lycopene is considered as a molecule which can inhibit lipid oxidation but not lipid concentration in the blood (Kong et al. 2010; Wang 2012). Therefore, the lipid-lowering ability of L-tug dark chocolate observed in our study is more likely to originate from an improved bioavailability of cocoa-derived bioactive compounds. As we have shown, microencapsulation of cocoa flavanols with lycopene and subsequent formation of coco-lycosomes enhanced the bioavailability of cocoa-bioactive compounds upon ingestion of the L-tug formulation of dark chocolate (Petyaev 2013). Moreover, recently the lycosome microencapsulation technology was successfully applied for other hydrophobic nutraceuticals with poor absorption rate — resveratrol, whey protein peptides (Petyaev et al. 2012; Bashmakov et al. 2014). Although the mechanism behind the lipid-lowering effect of L-tug chocolate remains elusive and needs to be investigated, the dark chocolate formulations with effect on lipid profile may be extremely helpful for the prevention of cardiovascular disease. Besides that, dark chocolate with an enhanced bioavailability of cocoa flavanols has a great promise as functional food. Most of the cardiovascular effects of dark chocolate are conditional and develop in a dose-dependent manner (Davison et al. 2010). It has been estimated that the blood pressure-lowering effect of cocoa flavanols requires at least 1052 mg of flavanols to be consumed (Zomer et al. 2012). Reduction in cardiovascular events reportedly may require daily consumption of cocoa polyphenols equivalent to up to 100 g of dark chocolate which roughly translates into a 500-calorie addition to daily calorific intake (Zomer et al. 2012). Therefore, besides new health applications, the development of a new formulation of dark chocolate with enhanced biological activity may help to minimize recommended daily amount of dark chocolate intake and reduce caloric intake without compromising on the health benefits of dark chocolate consumption.

In conclusion, our study shows that daily intake of lycopene-containing (L-tug) lycosome formulation of dark chocolate by healthy prehypertensive individuals with borderline-to-moderate hypercholesterolemia reduces the systemic blood pressure, as well as the total cholesterol and triglycerides in serum after the 1 month observational period. Similar amounts of dark chocolate and lycopene ingested as two separate formulations as well as consumption of dark chocolate alone do not affect serum lipids and reduce the systemic blood pressure at the conditions used in a less significant manner as compared to lycopene-containing dark chocolate.
